# Medical Defence

**Published:** 1911-03-18

**Authors:** 


### MEDICAL DEFENCE.

On not a few occasions The Hospital has urged upon all its professional readers the duty which they owe to themselves and their families to protect their pockets and reputation by joining one or other of the excellent defence Societies. As far as England is concerned there are two of these in the field: the Medical Defence Union, and the London and Counties Medical Protection Society. Notable instances of the value of both these Societies have recently been before the public: for the Defence Union conducted (and paid for) the successful defence of Mr. Ryall against a blackmailing action for negligence, and the London and Counties performed a like service at the end of February for Mr. Gwatkin, a dentist at Brighton, who was accused of dislocating a jaw while performing extraction. There have always been minor differences between the constitution and practice of these two Societies, though it is pleasant to be able to add that they both perform their functions in a manner beyond criticism. Thus the Defence Union does not admit dental members, who are therefore driven into the fold of the smaller Society. Another divergence of principle has now just been accomplished. By raising the subscription from ten shillings to a sovereign, the London and Counties has added to the benefits already afforded to its members by undertaking to pay any damages and costs awarded against its members up to the sum of £2,000 in any one year; and it will insure against any further loss of the like kind up to an additional £20,000 in any one year. This protection against adverse verdicts has hitherto been afforded, in both Societies, only by a separate contract between each member and an insurance company; for it has been thought that a judge or jury might conceivably take the view that such an extreme measure of protection is contrary to public policy. We have already, on a former occasion, combated that view, but it is one which a court of law in this country might conceivably take. It is now evident that the London and Counties Society has decided to risk it, and to risk also the financial consequences of an adverse verdict against a member. Such verdicts are so rare, and the reserve funds of the Societies are growing so rapidly, that the risk is one which may quite legitimately be taken. Still, the more cautious policy of the Medical Defence Union in holding aloof from a similar venture on behalf of its members is one for which there is a very great deal to be said. Time alone can show which plan will turn out best; meanwhile the two Societies afford two quite distinct measures of protection at two quite different premium rates, so that members of the profession can pay their money and take their choice. Once more we earnestly urge all to join one or other of them, or else some similar organisation such as that which flourishes across the Tweed. The successful handling of the rare cases such as the two lately before the courts constitutes in reality the least valuable part of their work. For every case thus fought to a finish there are scores or hundreds nipped in the bud at the outset by the *prestige* of the Societies and the experience of their advisers.

